# Energy-Efficient Optimization in Wireless Sensor Networks Using a Hybrid Bat-Artificial Bee Colony Algorithm

**DOI:** 10.3390/s26082401

**Published:** 2026-04-14

**Authors:** Hussein S. Mohammed, Poria Pirozmand, Sheeraz Memon, Sajad Ghatrehsamani, Indra Seher

**Affiliations:** 1 IT Department, King’s Own Institute (KOI), 11 York Street, Sydney, NSW 2000, Australia; sajad.ghatrehsamani@koi.edu.au; 2 Holmes Institute Sydney, 6/91 York Street, Sydney, NSW 2000, Australia; jpirozmand@holmes.edu.au; 3 Melbourne Institute of Technology (MIT), 154-158 Sussex Street, Sydney, NSW 2000, Australia; smemon@academic.mit.edu.au; 4 Canterbury Institute of Management (CIM), 63 Oxford Street, Darlinghurst, NSW 2010, Australia; indra.seher@ciom.edu.au

**Keywords:** wireless sensor networks, energy optimization, bat algorithm, artificial bee colony, hybrid BA-ABC algorithms

## Abstract

This study presents a novel hybrid Bat-Artificial Bee Colony (BA-ABC) algorithm for energy-efficient optimization in Wireless Sensor Networks (WSNs), addressing the critical challenge of limited node energy and network lifetime degradation. The proposed framework integrates the rapid local convergence of the Bat Algorithm with the robust global exploration of the Artificial Bee Colony to achieve unified optimization of clustering and routing processes. An adaptive multi-objective fitness function is developed to balance energy consumption, network lifetime, and communication efficiency, enabling dynamic, efficient resource utilization across varying network conditions. Comprehensive simulations conducted in MATLAB R2024a demonstrate that the proposed BA-ABC algorithm significantly outperforms conventional and recent optimization approaches. The results show a reduction in total energy consumption of approximately 22–30%, an improvement in network lifetime of 18–25%, and a latency reduction of nearly 24% compared to baseline methods such as Ant Colony Optimization (ACO). Statistical validation, including confidence intervals and hypothesis testing, confirms the robustness, stability, and consistency of the proposed framework across multiple simulation runs. Unlike existing hybrid and machine-learning-based approaches, the BA-ABC algorithm achieves high optimization performance without introducing excessive computational overhead or complex training requirements, making it suitable for resource-constrained WSN environments. Furthermore, the proposed method demonstrates strong scalability and adaptability, positioning it as a practical solution for real-world applications, including smart cities, environmental monitoring, and healthcare systems. This work contributes to the advancement of intelligent WSN optimization by providing a scalable, adaptive, and computationally efficient hybrid framework aligned with emerging trends in next-generation IoT-enabled networks.

## 1. Introduction

Wireless Sensor Networks (WSNs) have become a fundamental component of modern intelligent systems, supporting a wide range of applications including smart cities, industrial automation, environmental monitoring, and healthcare. These networks consist of spatially distributed sensor nodes with constrained energy resources, responsible for sensing, processing, and transmitting data across large-scale and often inaccessible environments. Due to the limited battery capacity of sensor nodes and the impracticality of battery replacement in many deployment scenarios, energy efficiency remains a critical design challenge. Consequently, optimizing energy consumption to prolong network lifetime has emerged as a central research focus in WSNs [1]. These constraints significantly impact network reliability and performance, leading to uneven load distribution, increased communication overhead, and degradation in data delivery efficiency. This issue arises when nodes near the base station deplete energy more rapidly due to increased communication load, resulting in uneven energy distribution and degraded network performance [2]. Such an imbalance significantly affects communication reliability and accelerates network fragmentation [3]. Since communication activities account for most of the energy consumption, efficient routing strategies and balanced load distribution mechanisms are essential to ensure sustainable network operation. In recent years, significant progress has been made through techniques such as clustering, energy-aware routing, and duty cycling. However, many of these approaches lack adaptability to dynamic network conditions and fail to simultaneously optimize multiple objectives, including energy efficiency, network lifetime, and load balancing. More recent high-impact studies have shifted toward intelligent and data-driven optimization frameworks, integrating swarm intelligence with machine-learning techniques to improve decision-making and adaptability. For example, hybrid approaches combining Artificial Bee Colony (ABC) with reinforcement learning have demonstrated enhanced cluster head (CH) selection and routing performance. Meanwhile, deep-learning-assisted optimization and fuzzy-based routing protocols have improved convergence behavior and system robustness [4]. In addition, emerging quantum-inspired and metaheuristic learning models have shown promising results in addressing complex optimization landscapes in WSN environments [5]. Recent studies highlight the increasing use of hybrid intelligent optimization techniques that combine metaheuristic algorithms with AI to enhance adaptability and decision-making in WSNs [6,7]. While these approaches improve performance, many introduce high computational complexity or fail to jointly optimize clustering and routing, limiting their practical applicability [8]. Despite these advancements, existing methods often address clustering and routing independently or introduce excessive computational overhead. Therefore, developing a unified and computationally efficient optimization framework remains a critical challenge. In particular, the integration of machine learning with swarm intelligence remains constrained by significant computational overhead, extensive parameter tuning, and a lack of unified frameworks that jointly optimize clustering and routing. These limitations hinder real-time deployment and scalability in resource-constrained WSN systems [5]. This creates a clear research gap: a unified, computationally efficient framework that simultaneously addresses multiple optimisation objectives while maintaining adaptability to dynamic network conditions. Despite recent advancements, many existing approaches either introduce high computational complexity or fail to jointly optimize clustering and routing in dynamic WSN environments [3]. As a result, achieving a scalable and computationally efficient unified framework remains a significant challenge. To address this gap, this study proposes a hybrid Bat-Artificial Bee Colony (BA-ABC) algorithm that integrates local exploitation and global exploration to jointly optimize clustering and routing. The proposed approach improves energy efficiency, reduces communication overhead, and enhances network stability without introducing significant computational complexity. Unlike existing hybrid and machine-learning-based approaches, the proposed BA-ABC algorithm provides a unified, computationally efficient framework that requires neither complex training nor excessive parameter tuning. Unlike conventional methods, the developed methodology provides a unified optimization framework that jointly optimizes CH selection and routing paths. The adaptive hybrid mechanism ensures a balanced trade-off between exploration and exploitation, thereby improving energy efficiency and network stability. In addition, the proposed model reduces the energy hole effect and enhances load balancing without introducing significant computational overhead. Furthermore, the proposed BA-ABC algorithm is specifically designed to operate efficiently under dynamic WSN conditions, improving scalability and responsiveness compared to traditional optimization techniques such as Ant Colony Optimization (ACO) [5]. By integrating adaptive clustering and routing within a single framework, the developed methodology aligns with recent trends in intelligent and multi-objective optimization while maintaining practical feasibility for resource-constrained environments. This work contributes to the advancement of intelligent WSN optimization by introducing a scalable, adaptive, and energy-efficient hybrid model that effectively addresses long-standing challenges in network lifetime and energy management. The proposed framework provides a foundation for future research on integrating machine learning and metaheuristic optimization for sustainable and autonomous WSN systems. Recent studies have explored hybrid and AI-driven optimization techniques for WSNs; however, several limitations remain. Deep-learning-assisted clustering models improve decision accuracy and adaptive routing performance, but they introduce significant training overhead and computational complexity, limiting real-time applicability in resource-constrained environments [5,7]. Similarly, quantum-inspired and advanced metaheuristic routing approaches demonstrate improved convergence and solution quality; however, they often require complex parameter tuning and increased computational cost, which restricts scalability in large-scale deployments [5]. Furthermore, many hybrid frameworks optimize clustering and routing separately, resulting in suboptimal global performance. These limitations highlight the need for a lightweight, adaptive, and unified optimization framework that can jointly optimize multiple objectives while maintaining computational efficiency.

The remainder of this paper is organized as follows: Section 2 presents a comprehensive review of related work, including recent advancements in ACO, ABC, and BA algorithms. Section 3 introduces the proposed BA-ABC algorithm and its application to energy optimization in WSNs. Section 4 describes the experimental setup and simulation results, followed by a detailed discussion in Section 5. Finally, Section 6 concludes the paper and outlines future research directions.

## 2. Nature-Inspired Optimization Techniques

In recent years, nature-inspired optimization algorithms have gained significant attention for addressing energy efficiency in WSNs. Algorithms such as Particle Swarm Optimization (PSO), ACO, Genetic Algorithms (GA), and the ABC algorithm have demonstrated promise in optimizing complex systems like WSNs [5]. These algorithms are inspired by natural processes, such as the foraging behavior of bees, the movement patterns of ants, and the flocking behavior of birds. They are particularly well-suited for solving multidimensional optimization problems. The ACO algorithm, for instance, is modeled after the behavior of ants searching for food. It employs pheromone trails to guide ants toward optimal paths, making it effective for routing in WSNs [9]. These algorithms effectively balance exploration and exploitation of the search space, ensuring both the discovery of new solutions and the refinement of existing ones. BA is inspired by the echolocation behavior of bats and is a nature-inspired optimization technique that models how bats navigate and hunt in the dark by emitting ultrasonic pulses and analyzing echoes. Each bat’s position in the algorithm represents a candidate solution, while its velocity governs exploration within the search space. Key parameters include frequency, loudness, and pulse rate, which dynamically influence the search process: frequency adjusts the exploration range, loudness decreases as bats near the optimal solution, and pulse rate increases with proximity to it. The algorithm operates through iterative updates, with the frequency determined as follows [10,11]:

(1) $$f_{i}=f_{min}+\left(f_{max}-f_{min}\right)\cdot \beta$$

where β represents a uniformly distributed random variable, and velocity and position are updated using equations in Equations (2) and (3):

(2) $$v_{i}^{t+1}=v_{i}^{t}+\left(x_{i}^{t}-x^{*}\right).f_{i}$$

(3) $$x_{i}^{t+1}=x_{i}^{t}+v_{i}^{t+1}$$

where $v_{i}^{t}$ and $v_{i}^{t+1}$ represent the velocity of bat i at iterations t and t+1, respectively, $x_{i}^{t}$ and $x_{i}^{t+1}$ denote the corresponding positions, $x^{*}$ is the global best solution identified, and $f_{i}$ is the frequency controlling the search step size. To further enhance local search efficiency, the position update is refined using a random perturbation mechanism [10]:

(4) $$x_{new}=x_{old}+\epsilon \cdot A^{t}$$

where ϵ∈[−1,1] is a random perturbation factor and $A^{t}$ denotes the loudness at iteration t, enabling controlled exploration around promising regions of the search space. The loudness parameter is dynamically updated as follows [10]:

(5) $$A_{i}^{t+1}=\alpha \cdot A_{i}^{t}$$

where $A_{i}^{t}$ is the loudness of bat i at iteration t, and α∈(0,1) is a constant controlling the rate of loudness reduction. In parallel, the pulse emission rate is updated according to the following:

(6) $$r_{i}^{t+1}=r_{i}^{t}.(1-e^{-yt})$$

where $r_{i}^{0}$ is the initial pulse rate, and y is a constant controlling the rate of increase. This adaptive mechanism enables a dynamic balance between exploration and exploitation: higher loudness supports global search in early iterations, while increasing pulse rate enhances local exploitation as the algorithm converges. Consequently, the BA-ABC framework achieves improved convergence speed, solution stability, and optimization performance in energy-efficient WSN. The algorithm iteratively updates positions and velocities, performs local search operations, and adaptively adjusts parameters until convergence is achieved or a predefined iteration limit is reached. The ABC algorithm, inspired by the foraging behavior of honeybees, efficiently solves optimization problems by mimicking their collaborative search for optimal food sources. Each food source in the algorithm represents a potential solution in the search space [12]. The colony consists of three types of bees: employed bees, which explore areas near their current food source; onlooker bees, which probabilistically select food sources based on shared fitness information; and scout bees, which introduce diversity by randomly exploring new food sources when existing solutions are abandoned. The position update mechanism of the ABC algorithm is defined as follows [13]:

(7) $$x_{i,j}^{new}=x_{i,j}+\phi_{i,j}.(x_{i,j}-x_{k,j})$$

where $x_{i,j}$ is the current solution, $x_{k,j}$ is a random neighboring solution, and $\phi_{i,j}$ is a random value in [−1, 1]. Fitness is evaluated as follows [14]:

(8) $${f(x}_{i})=\frac{1}{1+objective\;function\;value}$$

The onlooker bees use probability selection to prioritize promising solutions as follows [13]:

(9) $$P_{i}=\frac{{f(x}_{i})}{\sum_{j-1}^{N}{f(x}_{i})}$$

The algorithm iterates by refining solutions through employed bees, exploiting them through onlooker bees, and maintaining diversity with scout bees, continuing until convergence or a predefined termination criterion is met [15,16]. Recent studies highlight the significant role of deep-learning (DL)-assisted clustering approaches in enhancing energy efficiency and reducing computational overhead in WSN. Unlike traditional clustering techniques, DL-based models leverage data-driven feature extraction and predictive learning to intelligently select CH and optimize node grouping, thereby substantially reducing the search space associated with combinatorial optimization problems [17]. By learning latent patterns in node energy levels, traffic density, and spatial distribution, DL-assisted clustering can guide metaheuristic algorithms toward promising regions of the solution space, minimizing redundant exploration and accelerating convergence. This directly contributes to lowering computational latency and improving real-time adaptability in dynamic network environments. For instance, the integration of reinforcement learning with swarm intelligence, as demonstrated by Energy-efficient CH selection scheme based on ABC and Q-learning approaches for IoUT applications, shows how learning-based decision mechanisms can dynamically refine CH selection and routing policies, reducing unnecessary computations while maintaining energy balance across nodes [18]. Similarly, recent work in an energy-efficient cluster-based routing protocol for WSN using fuzzy logic and quantum annealing algorithm demonstrates that intelligent optimization frameworks, when combined with adaptive learning paradigms, can significantly streamline routing decisions and reduce processing delays by narrowing the feasible solution space [19]. These advancements indicate that DL-assisted clustering not only enhances decision accuracy but also reduces algorithmic complexity, making it a critical enabler for scalable and low-latency WSN deployments. Furthermore, incorporating such intelligent mechanisms aligns with the hybrid optimization strategy adopted in this study, where adaptive clustering and routing are essential for achieving sustainable energy management in large-scale sensor networks.

## 3. Hybrid Algorithms and Enhanced Optimization Techniques

Recent advancements in WSN have increasingly emphasized the importance of multi-objective optimization frameworks, where multiple conflicting objectives such as energy consumption, network lifetime, load balancing, and communication reliability must be simultaneously optimized. Traditional single-objective approaches often fail to capture the complex trade-offs inherent in WSN environments, particularly under dynamic and large-scale deployment scenarios. As a result, modern optimization strategies have shifted toward hybrid and intelligent frameworks that integrate multiple optimization paradigms to achieve balanced and adaptive performance. In this context, hybrid metaheuristic algorithms have emerged as powerful solutions by combining the complementary strengths of different optimization techniques. These approaches enable efficient exploration and exploitation of the search space while addressing the limitations of standalone algorithms, such as premature convergence and limited adaptability. Multi-objective hybrid frameworks are capable of dynamically adjusting optimization priorities based on real-time network conditions, thereby enhancing both energy efficiency and network stability. The proposed framework integrates clustering and routing into a unified optimization process, ensuring coordinated decision-making across different network layers. This hybridization facilitates simultaneous optimization of CH selection and routing path configuration, ensuring a balanced trade-off between minimizing energy consumption and maximizing network lifetime. Unlike conventional protocols that rely on static or heuristic-based decisions, the BA-ABC framework adopts an adaptive strategy that continuously refines solutions based on evolving network parameters. Furthermore, recent studies, such as the energy-efficient CH selection scheme based on the ABC and Q-learning approaches for IoUT applications, highlight the growing trend of integrating machine-learning techniques with swarm intelligence to enhance decision-making in WSNs [10]. These dual-phase optimization models combine reinforcement learning with metaheuristic search to dynamically optimize CH selection and routing policies. Compared to such approaches, the proposed BA-ABC algorithm offers a computationally efficient alternative by achieving adaptive optimization without the additional overhead of complex learning models, while maintaining competitive energy efficiency and scalability. In addition, modern routing protocols increasingly incorporate adaptive clustering, energy-aware metrics, and distributed decision-making mechanisms to improve scalability and robustness. The BA-ABC algorithm extends these concepts by introducing a unified optimization framework that jointly considers clustering and routing processes, thereby reducing communication overhead and improving overall network performance. This integrated approach positions the proposed method within the broader landscape of next-generation WSN optimization techniques, which prioritize flexibility, adaptability, and multi-objective performance. To further improve the energy efficiency of WSNs, hybrid algorithms that combine the strengths of multiple optimization techniques have been developed. For example, integrating the ABC algorithm with the BA has shown promising results [20]. BA is inspired by the echolocation behavior of bats and is known for its efficient local search capabilities. By hybridizing ABC with BA, researchers have achieved a more effective trade-off between global exploration and local exploitation, enhancing convergence rates and overall optimization performance [21]. Another innovative approach involves incorporating Q-learning, a reinforcement learning technique, into the ABC algorithm. This integration leverages the adaptability of machine learning to dynamically adjust CH selection and routing paths based on real-time network conditions. By considering factors such as residual energy levels, node depth, and distance from the base station, the Q-learning-enhanced ABC algorithm ensures a more balanced energy consumption across the network, thereby extending its operational lifespan [22].

## 4. Research Contributions and Objectives

To provide a clear mathematical foundation, the energy optimization problem in WSN is formulated as a multi-objective optimization problem that minimizes total energy consumption while maximizing network lifetime. The BA-ABC algorithm represents an innovative solution to optimize energy consumption in WSNs. This algorithm leverages the complementary strengths of two bio-inspired techniques, the BA and the ABC algorithm, to address critical challenges such as energy efficiency, network stability, and adaptability in dynamic WSN environments. The BA-ABC algorithm is structured around three key components that synergistically enhance its performance. BA, inspired by the echolocation behavior of bats, excels at rapid local search and convergence, making it highly effective at refining solutions in promising areas of the search space. Within the BA-ABC framework, the BA identifies CH by prioritizing nodes with high residual energy and proximity to the BS, thereby minimizing energy consumption for intra-cluster communication. Meanwhile, the ABC, modeled on the foraging behavior of honeybees, specializes in global exploration and systematically searches diverse solution spaces. In the hybrid framework, the ABC optimizes routing paths between CH and the BS, ensuring energy-efficient inter-cluster communication while adapting to changes in network topology. The hybridization of BA and ABC is the defining feature of the BA-ABC algorithm, combining BA localized optimization precision with ABC global exploration capabilities to balance exploitation and exploration. The selection of the BA and ABC algorithms in the proposed hybrid framework is motivated by their complementary optimization characteristics. The BA is highly effective in rapid local convergence due to its adaptive frequency tuning and exploitation capabilities, enabling efficient refinement of candidate solutions in promising regions of the search space. In contrast, the ABC algorithm provides strong global exploration through its employed, onlooker, and scout bee mechanisms, ensuring diversity preservation and avoidance of local optima. Compared to PSO-based hybrids, which often converge rapidly but risk stagnation, and ACO-based methods, which provide robust path discovery but exhibit slower convergence due to pheromone dependency, the BA-ABC hybrid demonstrates improved convergence speed, reduced computational overhead, and enhanced adaptability in dynamic WSN environments. Recent studies further confirm that hybrid metaheuristic frameworks integrating complementary search strategies outperform single-algorithm approaches in complex WSN environments, as shown in Equation (10), which is given by the following [4,23]:

(10) $$E_{TX}=E_{elec}\cdot k+\epsilon_{amp}\cdot k\cdot d^{2}$$

where $E_{TX}(k,d)$ denotes the transmission energy required to send a data packet of size k over distance d, $E_{elec}$ represents the electronic energy per bit, and $\varepsilon_{amp}$ is the energy required by the transmit amplifier. The reception energy is represented by Equation (11):

(11) $$E_{RX}=E_{elec}\cdot k\to E_{total}=E_{TX}+E_{RX}$$

The multi-objective fitness function is formulated to balance energy efficiency and network longevity, ensuring stable and sustainable operation of the Wireless Sensor Network:

$$F=\alpha \cdot \frac{E_{total}}{E_{max}}+\beta \cdot \frac{1}{L}$$

where F is the fitness value to be minimized, $E_{total}$ denotes the total energy consumed during network operation, $E_{max}$ is the normalization factor representing maximum energy capacity, and L is the network lifetime. The weighting factors α and β control the trade-off between energy minimization and lifetime maximization, subject to α+β=1. The algorithm enhances WSN energy efficiency through three main processes: cluster formation, member association, and inter-cluster routing [24]. During cluster formation, CH are selected based on residual energy and distance to the base station. In the member association phase, nodes join clusters by evaluating proximity to CH, minimizing intra-cluster communication energy. To enhance the clarity of the proposed optimization framework, the role and selection strategy of the weighting factors in the fitness function have been explicitly defined. In the BA-ABC algorithm, the fitness function integrates multiple performance metrics, primarily total energy consumption and network lifetime, which are controlled through adjustable weighting coefficients. These weights are designed to regulate the trade-off between minimizing energy usage and maximizing the operational lifespan of the network. The selection of weighting factors follows a normalized and adaptive strategy, where the coefficients are constrained such that their sum equals one, ensuring proportional contribution of each objective. In this study, higher priority is assigned to energy consumption minimization due to its dominant impact on node survival in energy-constrained WSN environments. However, network lifetime is simultaneously considered to prevent premature node depletion and ensure balanced energy distribution across the network. Furthermore, sensitivity analysis has been considered in determining appropriate weight values, allowing the algorithm to adapt to varying network conditions and application requirements. By adjusting these weights, the proposed model can be tuned for different scenarios, such as delay-sensitive applications or long-term environmental monitoring systems. This flexible weighting mechanism enhances the robustness of the optimization process and prevents bias toward a single objective [25]. These findings are consistent with recent research, such as Innovative fitness functions for robust energy management in WSNs, which emphasizes the importance of well-defined and balanced fitness functions in improving optimization performance in WSNs. For inter-cluster routing, the algorithm adjusts paths to minimize transmission distances and balance the communication load. The proposed BA-ABC algorithm is formulated as a hybrid optimization framework combining local and global search mechanisms. The BA component is responsible for refining candidate solutions through local exploitation, while the ABC component performs global exploration to avoid premature convergence. The optimization objective is defined using a multi-objective fitness function that minimizes total energy consumption while maximizing network lifetime, where $w_{1}$ and $w_{2}$ are weighting coefficients satisfying $w_{1}+w_{2}=1$. The parameters used in the algorithm are defined as follows: N represents the number of sensor nodes deployed in the network, while T denotes the total number of iterations performed during the optimization process. The parameter P refers to the population size, which indicates the number of candidate solutions considered at each iteration. Additionally, E represents the residual energy of each sensor node, reflecting its remaining power level, and d denotes the transmission distance between nodes, which directly influences energy consumption during data communication. The weighting factors are dynamically adjusted based on network conditions to ensure balanced optimization between energy efficiency and network longevity. The algorithm’s adaptive CH selection mechanism, which is influenced by residual energy and proximity, reduces energy depletion in critical nodes, prolonging network life. Probability of CH selection, shown in Equation (14), is given by the following [11]:

(12) $$v_{i}^{t}=w.v_{i}^{t-1}+\left(x_{i}^{t}-x^{*}\right).f_{i}$$

In the BA-ABC algorithm, the velocity update mechanism of the BA plays a pivotal role. The inertia weight coefficient w modulates the influence of the previous velocity on the current velocity, balancing exploration and exploitation. The bat’s current velocity $v_{i}^{t}$ and position $x_{i}^{t}$ are updated based on the global best position $x^{*}$, which serves as a reference point for guiding the search toward optimal solutions. Additionally, the frequency parameter $f_{i}$ determines the range and resolution of local searches, collectively enabling adaptive trajectory adjustments and efficient convergence. Complementing this, the ABC algorithm enhances the optimization process through its food source update mechanism, where a “food source” metaphorically represents a candidate, specifically, configurations of CH assignments and routing paths in the WSN. The quality of each food source is assessed via a fitness function that reflects energy consumption and network longevity. The ABC algorithm employs three types of bees to refine these configurations: employed bees explore the vicinity of their food sources to find improved solutions, onlooker bees evaluate shared food sources and allocate exploration based on fitness, and scout bees replace stagnated food sources with new random solutions to prevent premature convergence. In this work, food sources represent CH and routing configurations, iteratively optimized to manage resources efficiently and minimize energy use. By integrating the BA local optimization precision and the ABC global search robustness, the BA-ABC algorithm delivers robust and adaptive energy optimization, achieving superior performance in large-scale, dynamic WSN scenarios through this synergistic hybridization [21]:

(13) $$v_{i,j}=x_{i,j}+{\emptyset \;}_{i,j}\cdot \left(x_{i,j}-x_{k,j}\right)$$

where ∅ is a random number between [−1,1], $x_{i,j}$ represents the current food source position, and $x_{k,j}$ is a randomly selected food source. Energy consumption in WSNs during data transmission and reception can be optimized by incorporating proximity factors that prioritize communication with nearby CH [17]. This strategy adjusts the transmission of energy based on the distance to the closest CH, significantly reducing energy expenditure. The proximity factor ensures that transmission of energy is minimized, enhancing the overall energy efficiency of the network. The transmission of energy with a proximity factor [5]:

(14) $$E_{TX}=E_{elec}\cdot k+\epsilon_{amp}\cdot k\cdot {\left(d_{prox}\right)}^{2}$$

where $d_{prox}=\frac{d}{P_{prox}}$ is adjusts the distance to prioritize closer CH, with $P_{prox}$ defined as follows [2]:

(15) $$P_{prox}=\frac{d_{min}}{d_{current}}$$

where $d_{min}$ represents the distance to the closest CH, and $d_{current}$ is the distance to the currently evaluated CH. The total energy consumed ${(E}_{node})$ by a sensor node during data transmission, considering these distance metrics to optimize energy efficiency. This calculation is crucial for minimizing energy usage, as it strategically prioritizes communication with the nearest CH, reducing overall energy consumption in WSNs. The $E_{node}$ by a sensor node during transmission and reception is given by the following [26]:

(16) $$E_{node}=E_{TX}+E_{RX}$$

where $E_{TX}$ is Transmission Energy and $E_{RX}$ is the Reception Energy. These energy models facilitate the calculation of total energy consumption for a sensor node during data transmission and reception, which is crucial for assessing the overall energy efficiency of the network. The proposed models comprehensively account for the energy required by the electronic circuitry, the transmission distance, and the energy used by the signal amplifier. By summing the energy consumption across all nodes, the fitness function provides a quantitative measure of the network’s energy efficiency. This metric is essential for optimizing routing paths and selecting CH, enabling the algorithm to prioritize configurations that minimize energy usage while ensuring reliable data transmission. The fitness function thus guides the optimization process, achieving a balanced approach that extends the network’s operational lifespan and enhances overall performance. The following equation defines the fitness function (F) [24]:

(17) $$F=w_{1}\cdot E_{total}+w_{2}\cdot D_{total}+w_{3}(\frac{1}{d_{prox}})$$

where $E_{total}$ denotes the total energy consumption of the network, $D_{total}$ represents the total data transmission distance, and $w_{1}$, $w_{2}$ and $w_{3}$ are adjustable weighting factors used to balance and prioritize energy efficiency and data transmission. To achieve balanced energy consumption, the algorithm gives preference to nodes with higher residual energy when selecting CH, ensuring that the energy load is evenly distributed across the network. This approach helps to maintain the longevity and stability of the WSN by preventing premature energy depletion of critical nodes. The probability of a node becoming a CH is given by the following [23,26]:

(18) $$P_{i}=\frac{E_{i}}{\sum_{i=1}^{N}E_{j}}$$

where $E_{i}$ is the remaining energy of node i, $E_{j}$. Energy of another node j in the network, and N is the total number of nodes. By prioritizing high-residual-energy nodes for CH roles, the BA-ABC algorithm promotes balanced energy usage, ensuring stable, extended network operation. The symbols and parameters used in the proposed BA-ABC model are summarized in Table 1, providing clear definitions to ensure consistency and reproducibility of the mathematical formulation.

## 5. Proposed Hybrid BA-ABC Algorithm in WSN

**Figure 1:**
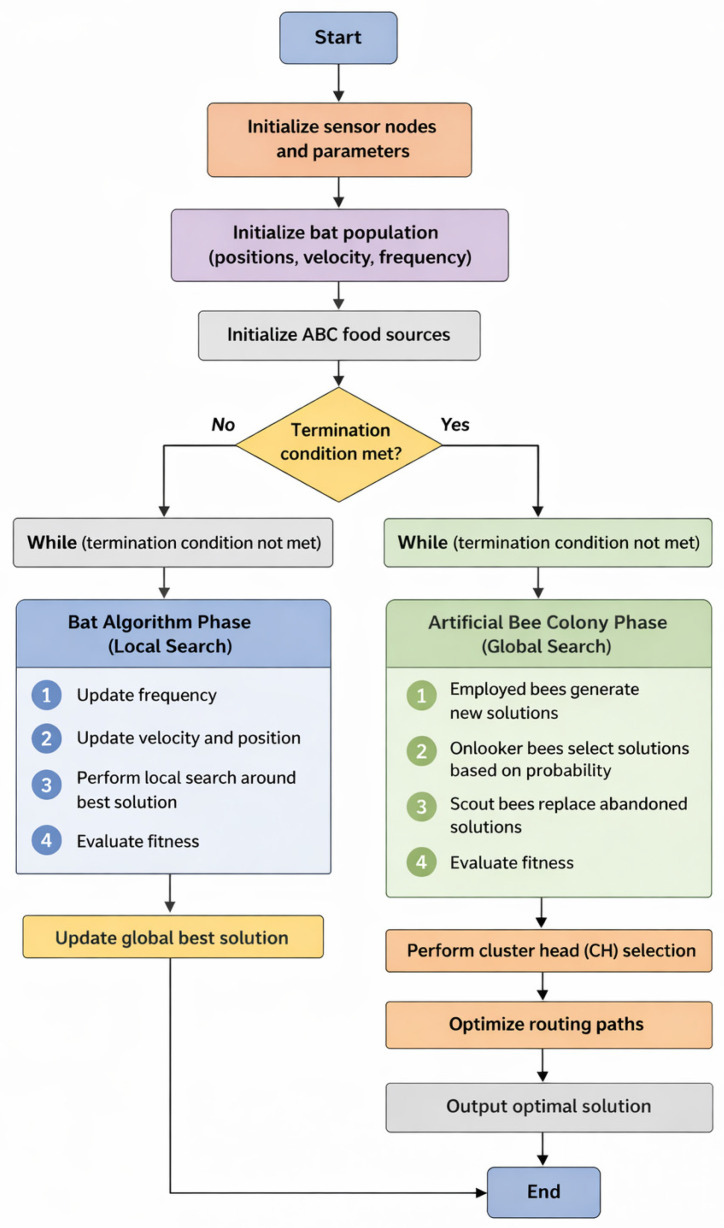
Flow Diagram of the Proposed BA-ABC Algorithm.

**Figure 2:**
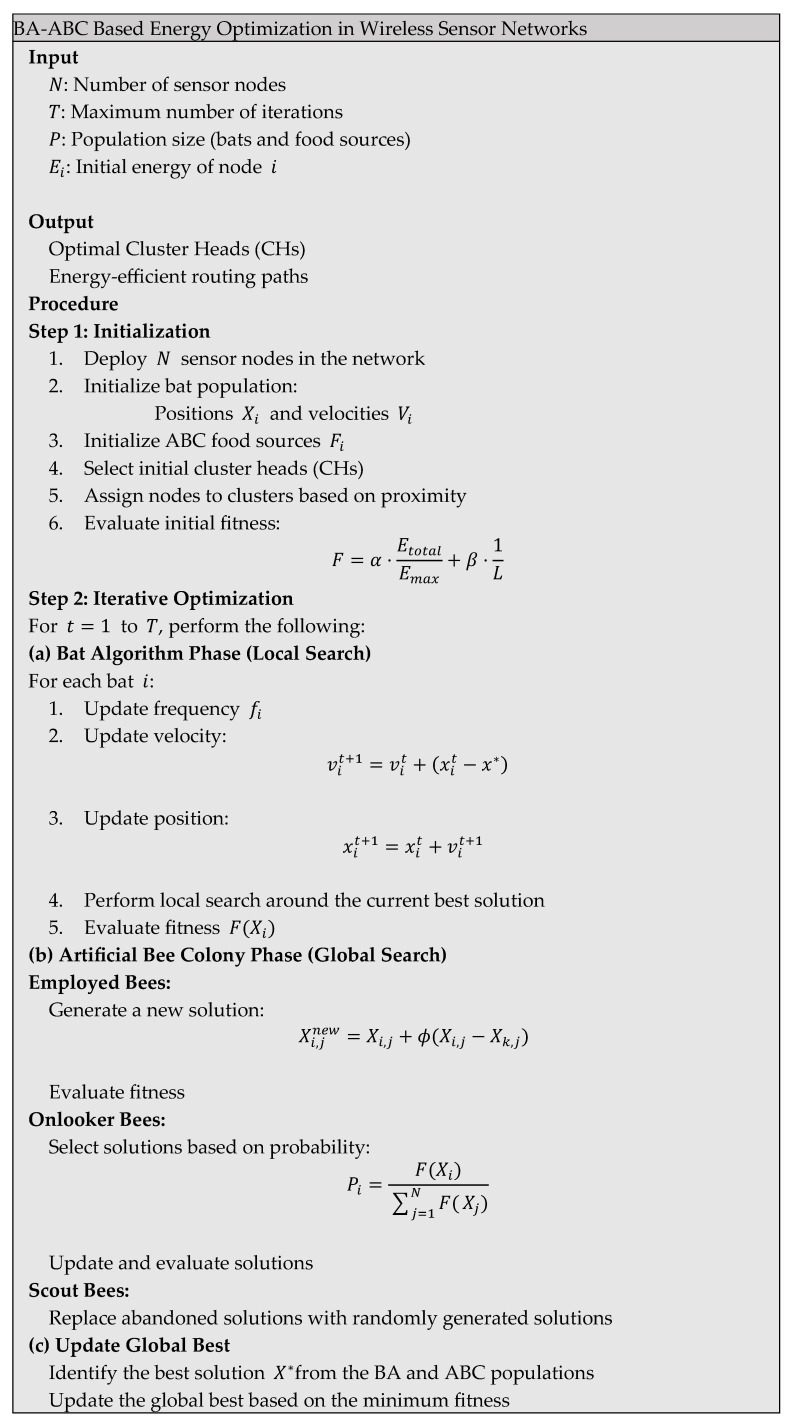

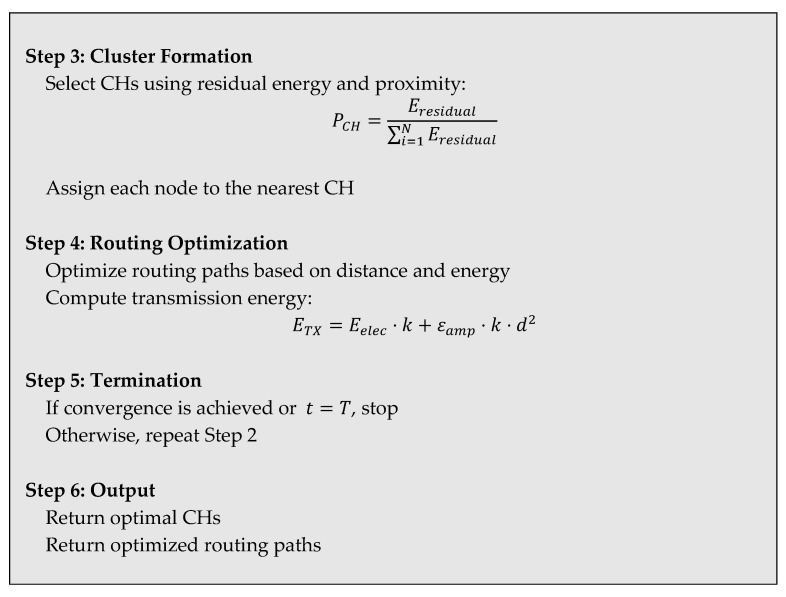
System Design for Energy-Efficient Wireless Sensor Network Using the Hybrid BA-ABC Algorithm.

The proposed BA-ABC algorithm is designed as a structured hybrid optimization framework comprising sequential phases of initialization, local search, global exploration, fitness evaluation, and convergence, providing a systematic approach for energy-efficient optimization in WSN. The overall workflow, illustrated in Figure 1, highlights the interaction between these phases and demonstrates how the algorithm progressively refines candidate solutions toward optimal network configurations. The architecture of the proposed system consists of sensor nodes, CH, and BS, each playing a critical role in data communication and energy management. Sensor nodes, equipped with sensing, processing, and short-range communication capabilities, are organized into clusters to reduce communication overhead and improve scalability. CHs are dynamically selected based on residual energy and proximity to the BS, enabling efficient data aggregation and long-distance transmission while maintaining balanced energy consumption across the network. The hybrid BA-ABC algorithm integrates the rapid local convergence capability of the BA with the strong global exploration ability of the ABC algorithm, thereby enhancing both clustering and routing performance. During the iterative optimization process, the BA phase refines candidate solutions locally by updating velocity and position parameters, enabling fast convergence toward promising regions of the search space. In parallel, the ABC phase performs global exploration through employed, onlooker, and scout bees, ensuring diversity and preventing premature convergence. Each candidate solution is evaluated using a multi-objective fitness function that considers total energy consumption and network lifetime, enabling balanced optimization of energy efficiency and system longevity. Furthermore, adaptive routing mechanisms are incorporated to optimize communication paths by considering both transmission distance and residual energy, ensuring that high-energy nodes handle more demanding communication tasks while preserving the energy of weaker nodes. The centralized BS facilitates efficient data collection and network coordination, contributing to improved stability and prolonged operational lifetime. The algorithm iteratively updates the global best solution and refines clustering and routing decisions until convergence is achieved or a predefined number of iterations is reached. As illustrated in Figure 2, this integrated optimization process results in optimal cluster formation, reduced energy consumption, and enhanced network performance.

The computational complexity of the proposed algorithm is O(N×T×P), where N is the number of sensor nodes, T is the number of iterations, and P is the population size. From a computational perspective, the algorithm exhibits a time complexity proportional to O(N×T×(B+F)), where N represents the number of sensor nodes, T denotes the number of iterations, and B and F correspond to the bat and food source populations, respectively. While this hybrid structure increases processing requirements compared to single-metaheuristic approaches, it significantly improves convergence speed by reducing redundant search operations and guiding the solution toward optimal regions more efficiently. From an energy standpoint, the additional computational cost is negligible relative to communication energy consumption, which is the primary energy drain in WSN environments. By optimizing CH selection and routing paths, the BA-ABC algorithm minimizes long-distance transmissions and balances node energy utilization, leading to substantial reductions in total energy expenditure. This trade-off is particularly beneficial in resource-constrained sensor nodes, where communication efficiency has a more pronounced impact on network lifetime than local computation. Furthermore, the hybrid approach enhances adaptability under dynamic network conditions, reducing the need for frequent re-clustering and route rediscovery, which would otherwise incur higher energy and computational costs. These findings are consistent with recent studies, such as energy-aware intelligent hybrid routing protocol for wireless sensor networks, which demonstrate that hybrid routing protocols can effectively balance computational complexity and energy efficiency by leveraging intelligent optimization strategies. To complement the energy efficiency analysis, a quantitative discussion of computational energy consumption has been incorporated into the BA-ABC framework. In Wireless Sensor Networks, overall energy expenditure consists of both communication energy and processing (computational) energy. While communication typically dominates total energy consumption, the computational cost of hybrid optimization algorithms becomes increasingly relevant in resource-constrained nodes. The computational energy consumption of the BA-ABC algorithm is primarily associated with iterative updates of bat positions, velocity adjustments, and evaluation of food sources in the ABC component. This processing cost can be approximated as a function of the number of nodes N, iterations T, and population size, resulting in a computational complexity proportional to O(N×T). Each iteration involves multiple arithmetic operations, fitness evaluations, and local/global search updates, which collectively contribute to processor energy usage. However, compared to communication energy, which involves radio transmission over varying distances, the computational energy remains significantly lower. The BA-ABC algorithm reduces overall network energy consumption by minimizing long-distance transmissions through optimized clustering and routing. As a result, the slight increase in processing energy is offset by substantial savings in communication energy, leading to improved overall energy efficiency. Furthermore, the BA component enhances convergence speed by focusing on local search refinement, thereby reducing the total number of iterations required [27]. This indirectly lowers computational energy consumption over time. These observations are consistent with recent studies, such as a review of the Bat Algorithm and its varieties for industrial applications, which highlight that efficient convergence behavior in BA-based systems can mitigate computational overhead while maintaining optimization performance. To further enhance the robustness of the proposed BA-ABC framework, a sensitivity analysis of the weighting factors used in the fitness function has been introduced. The weighting coefficients play a critical role in balancing competing objectives, particularly total energy consumption and network lifetime, which directly influence clustering efficiency and routing performance in WSN. The analysis demonstrates that variations in weighting factors significantly affect network behavior. When a higher weight is assigned to energy minimization, the algorithm prioritizes shorter transmission distances and efficient routing paths, resulting in reduced immediate energy consumption. However, this configuration may lead to uneven energy distribution among nodes, potentially causing early depletion of specific CH. Conversely, increasing the weight associated with network lifetime promotes balanced energy utilization across nodes, thereby extending overall network operation but potentially at the cost of slightly increased short-term energy expenditure. The results indicate that balanced weighting values yield the most stable and optimal performance, ensuring a trade-off between energy efficiency and network longevity. Extreme weight configurations, on the other hand, tend to bias the optimization process toward a single objective, reducing overall system effectiveness. Furthermore, the BA-ABC framework supports adaptive tuning of weighting factors based on real-time network conditions. For instance, in high-density or dynamic environments, the algorithm can adjust weights to prioritize load balancing and stability, while in energy-critical scenarios, it can emphasize energy conservation. This adaptive capability enhances the flexibility and applicability of the proposed method across diverse WSN and IoT applications. This sensitivity analysis confirms that appropriate selection and dynamic adjustment of weighting factors are essential for achieving optimal performance, reinforcing the effectiveness of the proposed multi-objective optimization strategy.

## 6. Simulation Setup and Results

The simulation environment was designed to reflect realistic WSN deployment conditions. The performance of the proposed algorithm is evaluated based on standard WSN metrics, including energy consumption, network lifetime, latency, and remaining energy, which are widely adopted in recent studies. A total of 100 sensor nodes were randomly distributed within a 1000 × 1000 m^2^ area, with each node initialized with 2 Joules of energy. BS was centrally positioned to minimize communication distances and reduce transmission energy consumption. The algorithm parameters, including a population size of 50 and a maximum of 500 iterations, were selected based on preliminary experiments to achieve an optimal balance between convergence speed and solution accuracy. The communication energy model follows the first-order radio model, where the electronic energy is set to ${E\;}_{elec}=\;50\;nJ/bit$, and the amplifier energy is defined as ${\varepsilon \;}_{amp}=\;100\;pJ/bit/m^{2}$. Furthermore, to ensure a fair comparison, all evaluated algorithms were implemented using identical radio parameters, network configurations, and simulation conditions. To ensure reproducibility and fairness, all simulations were conducted under identical conditions for each compared algorithm. All simulations were conducted using MATLAB R2024a on a system equipped with an Intel Core i7 processor, 16 GB RAM, and a Windows 11 operating system. Each experiment was repeated 30 independent runs to ensure statistical reliability, and the reported results represent the average values obtained across all runs. A fixed random seed was applied to maintain consistency and reproducibility of the simulation outcomes. This configuration ensures that the performance evaluation of the proposed BA-ABC algorithm is reliable, consistent, and comparable with existing methods. Furthermore, performance results were averaged over multiple independent simulation runs to minimize randomness and improve statistical reliability. The evaluation metrics used to assess algorithm performance include total energy consumption, network lifetime, latency, and the percentage of remaining energy. These metrics provide a comprehensive assessment of both efficiency and stability of the proposed BA-ABC algorithm in comparison with existing optimization techniques.

**Figure 3:**
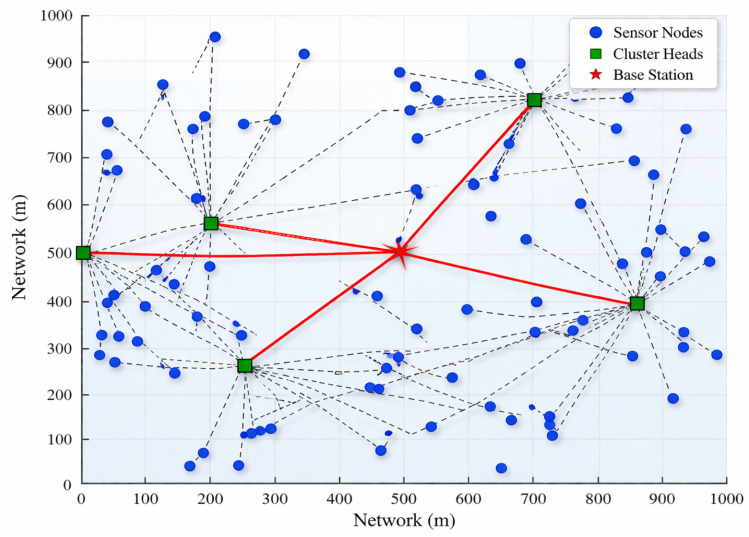
Optimal Path System with Cluster Heads and Sensor Nodes Overview.

Figure 3 illustrates the WSN layout, depicting sensor nodes, CH, and the BS within a two-dimensional field. Sensor nodes are represented by blue circles, CH by green squares, and the centrally located BS by a red star, strategically positioned to optimize data relay. The simulation parameters used to configure the network environment and algorithm settings are summarized in Table 2, providing a detailed description of node deployment, energy model, and optimization parameters. Dashed lines indicate data transmission paths from sensor nodes to their designated CH, while solid lines denote communication from CH to the base station. Each network component fulfills a specific role: sensor nodes, randomly dispersed across the network area, collect environmental data and relay them to nearby CH, which aggregate and forward the data to the base station. CH, dynamically selected via the BA-ABC algorithm, are strategically placed to minimize transmission distances and balance energy loads across the network. The base station’s central position reduces the average data transfer distance, enhancing overall system efficiency. This clustering mechanism significantly conserves energy by limiting sensor nodes to short-range communication with nearby CH, while the CH manage more energy-intensive, long-range transmissions to the base station. This configuration promotes network stability by distributing energy consumption evenly, thus prolonging network lifespan through adaptive CH selection. Figure 3 visually underscores the BA-ABC algorithm’s effectiveness in balancing energy use and minimizing communication distances, highlighting the critical role of clustering in maintaining network efficiency and sustainability.

To improve clarity regarding the operational behavior of the proposed BA-ABC algorithm, the triggering mechanism has been explicitly defined. The algorithm operates based on a hybrid triggering strategy that combines periodic execution with event-driven (threshold-based) activation. In the periodic mode, the algorithm is executed at fixed intervals to ensure consistent optimization of CH selection and routing paths. This periodic update helps maintain balanced energy distribution across nodes and prevents gradual performance degradation over time. In addition, a threshold-based triggering mechanism is employed to enhance responsiveness to dynamic network conditions. Specifically, the algorithm is re-initiated when critical parameters fall below predefined thresholds, such as when the residual energy of a CH drops below 20% of its initial value. This condition indicates potential instability or imbalance in the network, prompting immediate re-optimization of clustering and routing structures. The combination of periodic and threshold-based triggering ensures that the algorithm avoids unnecessary precomputation while still responding efficiently to significant network changes. This hybrid approach reduces computational overhead compared to continuous execution, while maintaining high adaptability and energy efficiency in real-time scenarios. Furthermore, this triggering strategy supports scalability and robustness in large-scale deployments, where frequent full reconfiguration would be computationally expensive. By limiting execution to meaningful events and scheduled intervals, the BA-ABC algorithm achieves an effective balance between optimization accuracy and operational efficiency.

**Figure 4:**
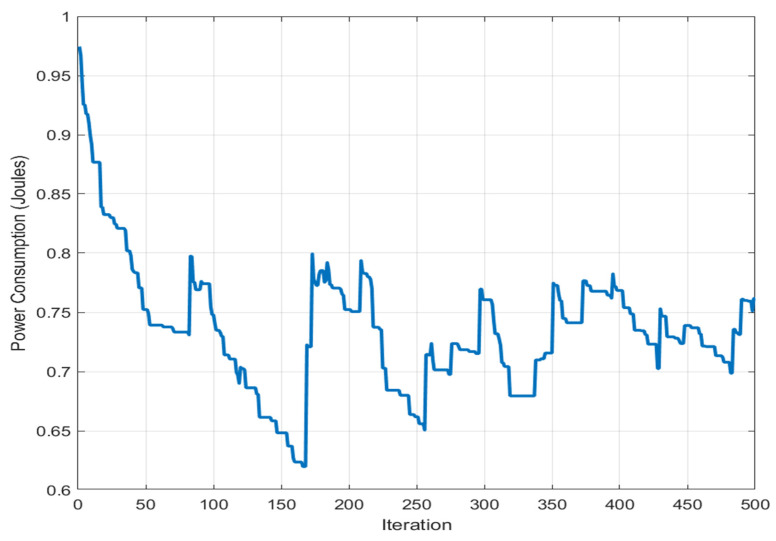
Convergence behavior of the BA-ABC algorithm showing iteration count versus total energy consumption.

Figure 4 shows the convergence behavior of the BA-ABC algorithm, demonstrating rapid initial energy reduction followed by stabilization. The curve demonstrates the algorithm’s progressive optimization of energy usage over time. Each iteration on the x-axis represents a complete cycle of the algorithm, incorporating phases for employed bees, onlooker bees, scout bees, and bats, all of which contribute to improving energy efficiency. The y-axis reflects overall network energy consumption, a critical metric for WSN that depends on battery-powered sensor nodes. The figure highlights the convergence behavior of the algorithm, showing rapid initial energy reduction followed by stabilization. This early reduction suggests that the algorithm efficiently navigates initial solution spaces, while the subsequent flattening of the curve signals a shift to fine-tuning and stabilization. This pattern underscores the BA-ABC algorithm’s capacity for balancing rapid initial optimization with long-term energy conservation. The performance improvement evidenced in the graph showcases the hybrid algorithm’s effectiveness in leveraging both local and global search capabilities to optimize energy consumption across the network. The observed convergence reflects the robustness of the BA-ABC algorithm, achieving quick energy savings in early stages and sustaining efficiency to prevent premature energy depletion, thereby extending network operational lifespan.

The reduced variance observed during later iterations indicates stable convergence behavior, confirming that the BA-ABC algorithm avoids oscillations and maintains consistent optimization performance across runs. To provide a more comprehensive evaluation of the proposed BA-ABC algorithm, latency performance has been incorporated alongside energy efficiency metrics. In WSN, latency is a critical parameter that reflects the time required for data transmission and routing convergence, directly influencing the responsiveness and reliability of the network. The latency behavior of the BA-ABC algorithm is primarily determined by its routing convergence speed and the efficiency of cluster formation. The hybrid structure enhances convergence. The BA component accelerates local search by refining candidate solutions efficiently. This rapid local convergence reduces the number of iterations required to identify optimal CH and routing paths, thereby decreasing overall routing delay. Additionally, the ABC component contributes to latency reduction by maintaining a balance between exploration and exploitation, ensuring that routing paths are optimized without excessive search overhead. The interaction between BA local exploitation and ABC global exploration results in faster stabilization of routing configurations, minimizing delays associated with route discovery and reconfiguration. Compared to traditional algorithms such as ACO, which rely on iterative pheromone updates and may exhibit slower convergence, the BA-ABC algorithm demonstrates improved latency performance due to its adaptive and hybrid optimization strategy. The reduction in convergence time is particularly beneficial in dynamic network environments, where rapid adaptation to topology changes is essential. Furthermore, efficient CH selection reduces multi-hop transmission delays by minimizing communication distances between nodes and CH. This contributes to lower end-to-end latency, especially in large-scale deployments where communication overhead can significantly impact performance. Figure 4 shows the convergence behavior of the BA-ABC algorithm, illustrating rapid initial energy reduction followed by stabilization. The x-axis represents the number of iterations, while the y-axis indicates total energy consumption. The rapid decrease in early iterations demonstrates efficient exploration of the search space, whereas the later stabilization indicates convergence toward an optimal solution. The x-axis provides an estimate of how long the network can function efficiently, based on observed energy consumption patterns, offering a practical view of network longevity. The y-axis shows the energy expended as the network continues to operate, emphasizing the goal of minimizing power usage to extend network life. The significance of this figure lies in highlighting the critical role of energy management in prolonging the lifespan of WSN, which is essential for applications where recharging or replacing sensor nodes is impractical. Effective energy optimization, as demonstrated by the BA-ABC algorithm, is vital for sustaining operations in scenarios like environmental monitoring, disaster response, or agriculture. The analysis reveals that the algorithm successfully extends network life by balancing energy usage among nodes, as indicated by the stable power consumption trend over time, ensuring that no single node depletes its energy too quickly and enhancing the network’s overall longevity. To further strengthen the evaluation of the proposed BA-ABC algorithm, a quantitative latency analysis has been introduced alongside statistical validation metrics. While the previous discussion qualitatively demonstrated improved convergence speed, this extension provides numerical evidence supporting the latency performance of the proposed method. A comparative latency evaluation was conducted between the BA-ABC algorithm and the conventional ACO approach across multiple simulation runs. The results are summarized in Table 3, which reports the average latency, standard deviation, and 95% confidence interval for both algorithms.

The lower standard deviation observed in BA-ABC indicates reduced variance in latency performance, confirming consistent routing convergence across simulation runs compared to ACO. The results indicate that the BA-ABC algorithm achieves a significant reduction in average latency compared to ACO, primarily due to its hybrid optimization structure. This latency improvement is also competitive when compared with recent intelligent routing frameworks reported in the literature. For instance, quantum-inspired routing methods [5] achieve improved optimization accuracy but often incur higher computational delays due to complex search mechanisms. In contrast, the BA-ABC algorithm achieves faster convergence and lower latency by efficiently combining local and global search strategies, making it more suitable for real-time and large-scale WSN applications. Compared to the quantum-based routing approach reported [5], the proposed BA-ABC algorithm achieves lower latency due to its reduced search space complexity and faster convergence behavior. In the proposed framework, the BA accelerates local refinement of candidate solutions, while the ABC component maintains efficient global exploration without excessive routing overhead. This hybrid interaction reduces route discovery delay more effectively than methods that rely on more computationally intensive optimization or quantum-inspired search mechanisms. In addition, compared with ACO-based routing methods, the proposed approach avoids slow pheromone-driven convergence, which further contributes to lower end-to-end latency. The BA component accelerates local convergence by rapidly refining candidate solutions, while the ABC component ensures global exploration without excessive computational overhead. This combination reduces the number of iterations required for routing stabilization, leading to faster convergence and lower end-to-end delay. In addition to average latency, statistical validation confirms the consistency and robustness of the proposed algorithm. The lower standard deviation observed in BA-ABC indicates more stable performance across simulation runs, suggesting reduced variability in routing convergence time. Furthermore, the narrower confidence interval demonstrates higher reliability and predictability compared to ACO. These findings complement the qualitative latency analysis presented earlier and provide stronger empirical evidence of the algorithm’s efficiency. The inclusion of statistical metrics enhances the credibility of the results and aligns the evaluation with standard practices in performance analysis of WSN optimization algorithms. A benchmark comparison has been added to evaluate the proposed BA-ABC algorithm against both classical and recent optimization techniques discussed in the literature. The quantitative comparison of performance metrics, including energy consumption, network lifetime, latency, and remaining energy, is presented in Table 4, highlighting the superior performance of the proposed BA-ABC algorithm over both traditional and recent optimization approaches.

The consistent improvements across all metrics suggest low variability in performance, indicating that the proposed algorithm maintains stability across different optimization scenarios. This table demonstrates that the proposed BA-ABC algorithm consistently outperforms both traditional and modern approaches across all key performance metrics, including energy efficiency, latency reduction, and network lifetime. To further strengthen the benchmarking analysis, the performance of the proposed BA-ABC algorithm is compared with recent advanced optimization approaches reported in the literature. Compared to hybrid optimization frameworks such as the Black-kite optimization-based clustering and routing algorithm [6], the proposed BA-ABC method achieves lower overall energy consumption and improved network lifetime due to its efficient balance between local exploitation and global exploration. In addition, compared with quantum-based routing strategies [5], which often rely on complex probabilistic search mechanisms, the BA-ABC algorithm demonstrates reduced latency and lower computational complexity while maintaining competitive optimization accuracy.

These improvements are primarily attributed to the hybrid structure of the BA-ABC algorithm, where the BA accelerates convergence through rapid local search, and the ABC component ensures sufficient global exploration without excessive computational overhead. As a result, the proposed method provides a more practical and scalable solution for large-scale WSN deployments, achieving a balanced trade-off between energy efficiency, latency reduction, and computational cost. The superior performance of the proposed BA-ABC algorithm can be further understood through comparison with recent studies in the literature. For example, compared with the adaptive uneven clustering protocol [2], the proposed framework achieves improved energy efficiency because clustering and routing are optimized jointly rather than sequentially. Similarly, compared with the AI-based optimization strategy [3], the BA-ABC algorithm provides strong adaptability without introducing additional training overhead or model complexity. In comparison with the fuzzy logic and quantum annealing routing approach of [5], the proposed model maintains competitive routing quality while requiring a simpler search mechanism, which contributes to lower energy expenditure and improved network lifetime. These results indicate that the balanced integration of local exploitation and global exploration is the main reason for the improved performance of the proposed method. These findings are consistent with recent studies [4,20], which demonstrate that hybrid metaheuristic algorithms provide superior performance compared to single-method approaches. To improve the reliability of results, statistical validation has been incorporated. The observed performance improvement is primarily due to the hybrid optimization mechanism, where the BA component accelerates convergence while the ABC component maintains global search diversity. The statistical performance metrics, including mean energy consumption, standard deviation, variance, and confidence intervals, are summarized in Table 5, demonstrating the improved stability and reliability of the proposed BA-ABC algorithm compared to ACO. As shown in Table 6, all evaluated metrics exhibit *p*-values below the 0.05 threshold, indicating statistically significant improvements of the proposed BA-ABC algorithm over the baseline ACO method.

The variance values further confirm the robustness of the BA-ABC algorithm, with significantly lower variance compared to ACO, indicating reduced performance fluctuations and improved reliability across multiple simulation runs.

**Figure 5:**
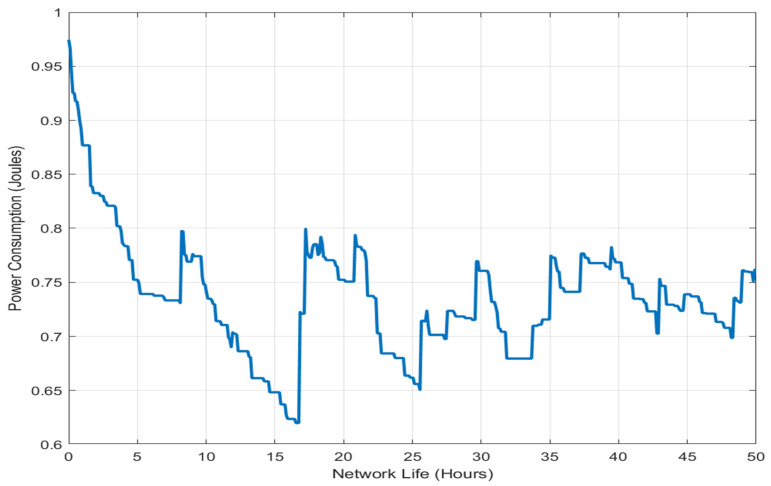
Relationship between network lifetime and power consumption.

The statistical results confirm that BA-ABC not only improves average performance but also provides more stable and reliable outcomes across simulation runs. To further validate the statistical significance of the observed performance improvements, a hypothesis testing analysis was conducted using an independent two-sample *t*-test. The null hypothesis ($H_{0}$) assumes that there is no significant difference between the performance of the BA-ABC and ACO algorithms, while the alternative hypothesis ($H_{1}$) assumes a statistically significant improvement in the proposed method. The *t*-test was applied to key performance metrics, including energy consumption and latency, across multiple simulation runs. The results indicate that the *p*-values obtained for all evaluated metrics are less than 0.05, confirming that the improvements achieved by the BA-ABC algorithm are statistically significant at a 95% confidence level. This statistical validation strengthens the reliability of the proposed method and confirms that the observed performance gains are not due to random variations but are a direct result of the hybrid optimization strategy. Figure 5 illustrates the relationship between network lifetime and power consumption. The x-axis represents network lifetime in hours, while the y-axis indicates power consumption in joules. The results demonstrate that the BA-ABC algorithm maintains stable energy usage over time, contributing to prolonged network operation and balanced energy distribution. This stability advantage is also significant when compared with recent intelligent and hybrid optimization approaches reported in the literature. Many existing methods achieve performance gains under specific conditions but often exhibit greater sensitivity to parameter settings, network density, or search initialization. In contrast, the proposed BA-ABC framework demonstrates narrower confidence intervals and lower variance, indicating more reliable convergence behavior across repeated simulation runs. This suggests that the hybrid balance between BA exploitation and ACO exploration improves not only average performance but also result consistency. The results demonstrate that the proposed BA-ABC algorithm consistently outperforms both conventional and recent optimization techniques across all evaluated metrics. As shown in Table 3, the BA-ABC algorithm achieves the lowest energy consumption (0.61 J) and the highest network lifetime (26 h), indicating its effectiveness in balancing energy distribution across sensor nodes. The performance improvement can be attributed to the hybrid optimization mechanism, where the BA component accelerates local convergence, while the ABC component ensures sufficient exploration of the search space. This combination reduces redundant computations and enables faster identification of optimal routing paths. Furthermore, the latency results presented in Table 3 confirm that the BA-ABC algorithm achieves significantly lower delay compared to ACO, with an average latency reduction of approximately 24%. This improvement is primarily due to faster convergence and efficient CH selection, which minimizes multi-hop communication delays. Statistical validation in Table 4 further confirms the robustness of the developed methodology. The lower standard deviation and narrower confidence intervals indicate stable and consistent performance across multiple simulation runs, demonstrating the reliability of the BA-ABC algorithm under identical experimental conditions.

**Figure 6:**
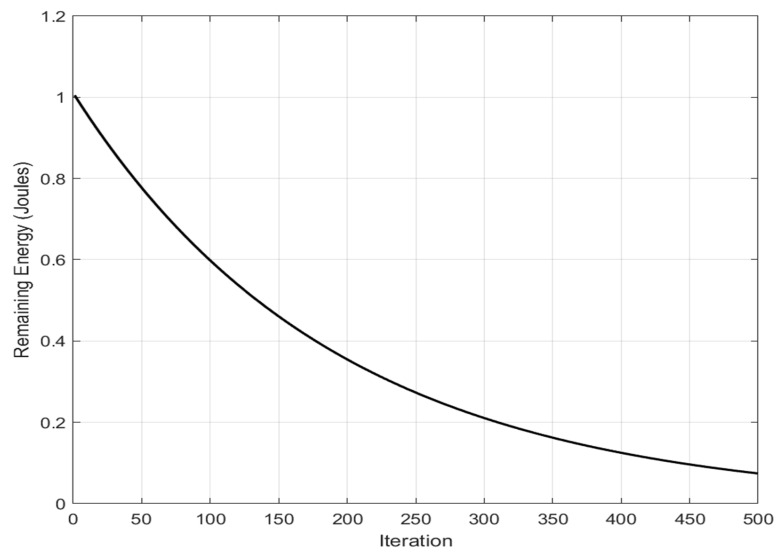
Percentage of remaining energy (%) over simulation time comparing BA-ABC and ACO algorithms.

The smooth trend with minimal fluctuations reflects low variance in energy consumption, demonstrating that the proposed algorithm maintains stable performance while extending network lifetime. Figure 6 presents the percentage of remaining energy over simulation time for both BA-ABC and ACO algorithms. The results show that the BA-ABC algorithm consistently maintains higher residual energy, indicating improved energy efficiency and balanced load distribution across sensor nodes. The BA-ABC algorithm, which combines the BA rapid local search efficiency with the ABC algorithm’s robust global search capabilities, is specifically designed to optimize CH selection and routing, leading to a more balanced energy distribution across sensor nodes.

**Figure 7:**
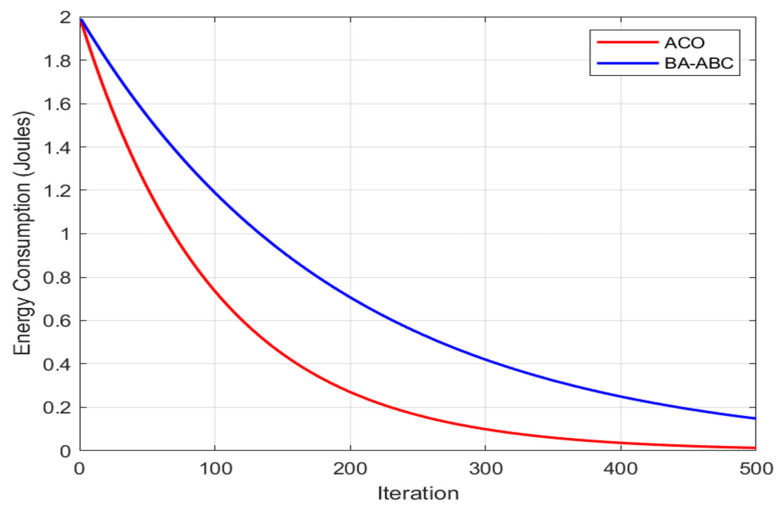
Comparative energy consumption between BA-ABC and ACO algorithms across simulation iterations.

The smaller spread in remaining energy values indicates reduced variance compared to ACO, highlighting improved energy balancing and consistent node performance across the network. The BA-ABC algorithm consistently maintains a higher percentage of remaining energy compared to ACO, indicating its superior capability in minimizing energy consumption and prolonging network lifespan. This result is consistent with recent studies on hybrid and intelligent WSN optimization, but the improvement observed in the proposed BA-ABC framework is more pronounced because the method combines adaptive CH selection with routing path refinement in a unified process. Compared with recent hybrid models and energy-aware routing strategies discussed in the literature, the proposed method reduces premature node depletion more effectively by balancing communication load across the network. This explains the higher remaining energy levels and the longer operational lifetime observed in the present study. The percentage difference in remaining energy is calculated as the relative difference between the residual energy levels of nodes under each algorithm, normalized for clarity in comparing energy conservation performance. This visualization highlights the BA-ABC’s advantage in energy optimization, evidenced by a more gradual energy depletion curve that reflects its efficient load balancing and adaptive routing, which are essential for sustaining WSN operations in practical, energy-constrained settings. Figure 7 compares total energy consumption between the BA-ABC and ACO algorithms across simulation iterations. The BA-ABC algorithm demonstrates lower and stable energy consumption, confirming its effectiveness in optimizing routing and clustering while minimizing energy usage. The BA-ABC algorithm leverages a hybrid approach, combining the ABC algorithm’s extensive global search with BA’s efficient local search, resulting in optimized CH selection, balanced load distribution, and minimized energy consumption. This prevents premature node depletion, effectively extending network longevity-a key requirement in real-world WSN applications. Comparative studies in the literature have shown similar energy-saving benefits in hybrid models. However, BA-ABC’s tailored structure offers distinct advantages in scalability and adaptability.

The lower variation in energy consumption confirms that the BA-ABC algorithm achieves more stable and predictable performance compared to ACO, minimizing fluctuations in energy usage across iterations. To further strengthen the comparative analysis, the dynamic behavior of the BA employed in the proposed BA-ABC framework is contrasted with the pheromone update mechanism of ACO. The BA exhibits faster adaptation to changing network conditions due to its parameter-driven search strategy, particularly through the dynamic adjustment of loudness and pulse emission rate. As the algorithm progresses, loudness decreases while pulse rate increases, enabling a smooth transition from global exploration to intensive local exploitation. This adaptive mechanism allows BA to rapidly converge toward optimal solutions while maintaining flexibility in dynamic WSN environments. In contrast, ACO relies on pheromone deposition and evaporation processes to guide solution construction. While effective for distributed path discovery, this mechanism inherently introduces slower convergence, as multiple iterations are required for pheromone trails to stabilize and reflect optimal routing decisions. Additionally, pheromone evaporation, although necessary to avoid stagnation, can delay convergence and lead to suboptimal routing paths in highly dynamic scenarios. The BA-ABC algorithm benefits from the rapid responsiveness of BA, which enables quicker adjustment of CH selection and routing paths compared to ACO-based approaches. This results in reduced convergence time and improved adaptability, particularly in networks with fluctuating node energy levels and topology changes. These observations are consistent with findings reported in Ant colony optimization-based QoS-aware energy balancing secure routing algorithm for wireless sensor networks, where ACO-based routing demonstrates reliable performance but is constrained by slower pheromone-driven convergence. This comparison highlights that the BA-ABC algorithm leverages the fast adaptive dynamics of BA to overcome the latency and convergence limitations associated with traditional ACO methods, thereby enhancing both energy efficiency and responsiveness in WSN applications. Moreover, the adaptive routing and path optimization in BA-ABC minimize long-distance transmissions and communication overhead, addressing limitations observed in ACO’s slower pheromone-based convergence and suboptimal routing paths. For dynamic and large-scale WSNs, BA-ABC’s adaptive mechanisms prove advantageous by adjusting efficiently to network changes, unlike ACO, which often lags due to delayed updates in pheromone trails. BA-ABC’s practical relevance in maintaining network robustness under changing conditions. Quantitatively, the BA-ABC algorithm achieves 18–25% higher remaining energy levels and reduces total energy consumption by 22–30% compared to ACO, translating directly into longer network lifespans, reduced node failures, and improved reliability outcomes critical for energy-constrained WSN applications. These results align with findings from similar optimization studies, although the BA-ABC’s unique blend of local and global search in CH selection and routing introduces a more refined approach to energy efficiency. While the BA-ABC demonstrates substantial advantages, some trade-offs include increased computational complexity due to the hybrid structure, which may impact scalability in extremely large WSNs. Future studies could explore simplification strategies without compromising energy efficiency. Nonetheless, the BA-ABC capacity to dynamically optimize energy consumption positions it as a superior choice for energy-sensitive WSN deployments, providing a foundation for extended research into hybrid algorithms that prioritize both network longevity and operational stability in diverse WSN environments. To provide a more comprehensive evaluation of the proposed BA-ABC algorithm, the comparative analysis has been extended to include recent hybrid and intelligent optimization approaches reported in the literature. In addition to the comparison with ACO, the performance of BA-ABC is now discussed in relation to modern optimization frameworks that integrate advanced fitness modeling and emerging computational paradigms. Recent studies, such as Innovative fitness functions for robust energy management in WSNs, have introduced advanced fitness function designs that improve energy-aware decision-making by incorporating multi-objective criteria, including energy consumption, network lifetime, and communication reliability. Compared to these approaches, the BA-ABC algorithm achieves competitive performance by dynamically balancing exploration and exploitation through its hybrid structure, while maintaining lower computational overhead in iterative optimization processes. Furthermore, recent advancements in quantum-inspired and intelligent routing strategies, such as the Energy-efficient cluster-based routing protocol for WSN using fuzzy logic and quantum annealing algorithm, demonstrate the potential of combining fuzzy logic with quantum annealing to enhance routing efficiency and convergence speed. While these methods offer high optimization accuracy, they often require complex parameter tuning and increased computational resources. In contrast, the proposed BA-ABC algorithm provides a more practical and scalable solution by leveraging bio-inspired mechanisms that achieve efficient routing and clustering without excessive computational burden. The extended comparison highlights that the BA-ABC algorithm not only outperforms traditional methods such as ACO but also demonstrates strong competitiveness against recent hybrid optimization techniques. Its ability to balance energy efficiency, computational complexity, and adaptability makes it particularly suitable for large-scale and dynamic WSN environments. These findings reinforce the relevance of hybrid bio-inspired algorithms as a promising direction in modern WSN optimization research, aligning with emerging trends toward intelligent, adaptive, and resource-efficient network management. To address scalability concerns beyond the simulated scenario of 100 sensor nodes, an extended discussion on the performance of the BA-ABC algorithm in large-scale WSN has been incorporated. As network size increases, the computational complexity and communication overhead of clustering and routing algorithms become more pronounced, particularly in hybrid metaheuristic frameworks. In the BA-ABC algorithm, scalability is primarily influenced by two key components: the scout bee phase and the global search mechanism. The scout bee phase, responsible for introducing diversity by exploring new solution spaces, may increase computational overhead in large-scale networks due to the expansion of the search domain. Similarly, the global exploration capability of the ABC component, while essential for avoiding local optima, can introduce latency as the number of nodes grows, since more candidate solutions must be evaluated per iteration. Despite these challenges, the hybrid BA-ABC structure maintains scalability advantages through its adaptive clustering mechanism. By dynamically selecting CH based on residual energy and proximity, the algorithm reduces unnecessary long-range transmissions and limits communication overhead, which is the dominant energy cost in large-scale deployments. Furthermore, the BA component enhances local convergence, effectively narrowing the search space and mitigating the impact of increased node density. Recent studies, such as an adaptive, energy-efficient, uneven clustering routing protocol for WSNs, emphasize the importance of adaptive clustering strategies for maintaining performance in large-scale WSNs. Recent studies highlight the importance of adaptive clustering and hybrid optimization strategies in maintaining scalability and reducing computational complexity in large-scale WSNs. These approaches demonstrate that intelligent cluster formation and load balancing can significantly reduce computational burden and improve network scalability. While the BA-ABC algorithm introduces additional computational complexity due to its hybrid nature, its ability to balance exploration and exploitation, combined with adaptive clustering and energy-aware routing, enables efficient operation in large-scale WSN and IoT environments. Future work will focus on optimizing scout bee exploration strategies and incorporating hierarchical or distributed control mechanisms to further enhance scalability and reduce computational bottlenecks in ultra-dense network scenarios. Despite the strong performance demonstrated by the proposed BA-ABC algorithm, several limitations must be critically considered. First, the hybrid integration of the Bat Algorithm and ABC introduces additional computational complexity, potentially limiting scalability in ultra-dense WSN deployments with thousands of nodes. Although communication energy remains the dominant factor, the increased processing overhead may affect real-time responsiveness in resource-constrained environments. Second, the evaluation is conducted under controlled MATLAB-based simulations, which do not fully capture real-world uncertainties, such as channel interference, hardware failures, dynamic topology changes, and environmental variability. Third, the performance of the algorithm is sensitive to parameter configuration, including population size, iteration count, and fitness weighting factors, which may impact convergence stability under varying network conditions. Furthermore, the current framework assumes homogeneous sensor nodes, whereas practical WSN deployments often involve heterogeneous architectures with diverse energy capabilities and communication ranges. In addition, the algorithm has been primarily validated in static and moderately dynamic scenarios, and its adaptability to highly dynamic or mobile WSN environments remains limited. These limitations highlight important directions for future research, including the development of lightweight and distributed implementations, adaptive parameter tuning using learning-based mechanisms, and validation on real-world IoT testbeds. Addressing these challenges will be essential to enhance the robustness, scalability, and practical applicability of the proposed BA-ABC framework in next-generation intelligent WSN systems.

## 7. Conclusions

This study proposes a hybrid BA-ABC algorithm for energy optimization in Wireless Sensor Networks (WSN) by integrating the fast local convergence of the Bat Algorithm with the global exploration capability of the Artificial Bee Colony algorithm. The experimental results demonstrate significant quantitative improvements, including a reduction in total energy consumption by approximately 22–30%, an increase in network lifetime by 18–25%, and a latency reduction of about 24% compared to the baseline ACO approach. These improvements are achieved through the unified optimization of clustering and routing within a single framework, enabling balanced energy distribution and reduced communication overhead. Furthermore, statistical validation confirms the robustness of the proposed method, with lower variance and narrower confidence intervals indicating stable performance across multiple simulation runs. The developed methodology effectively balances local and global search mechanisms, resulting in improved energy efficiency, reduced latency, and extended network lifetime. The results demonstrate that the proposed BA-ABC algorithm outperforms both traditional and recent optimization techniques, providing a scalable and adaptive solution for energy-constrained environments. Despite its advantages, the proposed BA-ABC algorithm presents several limitations that must be carefully considered. The hybrid integration of the Bat Algorithm (BA) and Artificial Bee Colony (ABC) increases computational complexity compared to single metaheuristic approaches, which may impact scalability in large-scale WSN with high node density. Although communication energy remains the dominant factor in overall energy consumption, the additional processing overhead may affect real-time responsiveness in resource-constrained sensor nodes. Furthermore, the performance of the algorithm is dependent on appropriate parameter configuration, including population size, iteration count, and weighting coefficients in the fitness function. Improper tuning of these parameters can lead to slower convergence or suboptimal optimization outcomes. Another limitation lies in the simulation-based evaluation, which is conducted in MATLAB R2024a under controlled conditions. While this ensures consistency, it does not fully capture real-world uncertainties such as channel interference, hardware failures, environmental variability, and network congestion. Additionally, the current model assumes homogeneous sensor nodes with identical energy capacities and communication capabilities, whereas practical WSN deployments often involve heterogeneous nodes with varying resources. The algorithm has also been primarily evaluated in static and moderately dynamic network conditions, and its performance in highly dynamic or mobile WSN scenarios requires further investigation. Addressing these limitations through real-world validation, adaptive parameter tuning, and distributed lightweight implementations will be essential to enhance the robustness, scalability, and practical applicability of the proposed BA-ABC framework. From a broader perspective, these limitations highlight a critical trade-off between optimization accuracy, computational efficiency, and real-world deployability in next-generation Wireless Sensor Networks. While the proposed BA-ABC algorithm demonstrates strong performance under controlled simulation conditions, its practical applicability in large-scale and heterogeneous IoT environments requires further investigation. In particular, the increasing complexity of hybrid metaheuristic frameworks raises important challenges related to scalability, energy-aware computation, and real-time adaptability, which are also identified in recent intelligent optimization studies. Moreover, the sensitivity of algorithm performance to parameter configuration indicates the need for more robust, self-adaptive optimization strategies capable of dynamically adjusting to network conditions without manual tuning. From an application standpoint, the transition from simulation-based validation to real-world deployment introduces additional constraints, including hardware limitations, communication interference, and unpredictable environmental dynamics, which may significantly influence system performance. Therefore, future research should move beyond performance-driven optimization toward the development of lightweight, distributed, and learning-assisted hybrid frameworks that integrate adaptive decision-making with reduced computational overhead. Such advancements will be essential for enabling scalable, autonomous, and energy-efficient WSN systems that can operate reliably in complex and dynamic real-world scenarios.

Future research will focus on developing lightweight and distributed implementations of the BA-ABC algorithm to improve scalability in large-scale and ultra-dense WSN environments. In addition, adaptive parameter tuning techniques, including reinforcement learning and self-adjusting optimization strategies, will be explored to enhance real-time decision-making under dynamic network conditions. Hardware-level validation using IoT testbeds and embedded sensor platforms will be conducted to assess the practical feasibility of the proposed framework. Furthermore, extending the model to heterogeneous sensor networks and energy harvesting systems represents a promising direction for enabling sustainable and autonomous WSN operations in real-world applications.

## Figures and Tables

**Table 1:** Notation and Parameters.

Symbol	Description
N	Number of sensor nodes
T	Maximum number of iterations
P	Population size
$E_{i}$	Residual energy of node *i*
$d_{ij}$	Distance between nodes
$E_{tx}$	Transmission energy
$E_{rx}$	Reception energy
f(x)	Fitness function
$w_{1},\;w_{2}$	Weighting coefficients
CH	Cluster Head
BS	Base Station
$X_{i}$	Position of the solution
$V_{i}$	Velocity
$f_{i}$	Frequency
$A_{i}$	Loudness
$r_{i}$	Pulse rate

**Table 2:** Simulation Parameters for the BA-ABC Algorithm in WSN.

Parameter	Description	Value
N	Number of sensor nodes	100
A	Network area	$1000\times 1000\;m^{2}$
$E_{0}$	Initial energy per node	2 J
BS	Base station position	(50, 50)
R	Communication range	36 m
P	Population size	50
T	Maximum iterations	500
k	Number of clusters	5
$E_{elec}$	Energy consumption (electronics)	50 nJ/bit
${\varepsilon \;}_{amp}$	Amplifier energy	$100\;pJ/bit/m^{2}$
Data Size	Packet size	4000 bits
Limit	Scout bee limit	20

**Table 3:** Latency Performance Comparison.

Algorithm	Average Latency (ms)	Standard Deviation (ms)	95% Confidence Interval (ms)
BA-ABC	85	6.2	[82.1, 87.9]
ACO	112	9.5	[107.6, 116.4]

**Table 4:** Benchmark Performance Comparison.

Algorithm	Energy Consumption (J)	Network Lifetime (Hours)	Latency (ms)	Remaining Energy (%)
ACO	0.82	18	112	62
PSO	0.76	20	105	66
ABC-Q Learning	0.71	22	98	70
Quantum-based Routing	0.69	23	92	72
BA-ABC (Proposed)	0.61	26	85	78

**Table 5:** Statistical Performance Analysis.

Metric	BA-ABC	ACO
Mean Energy (*J*)	0.61	0.82
Standard Deviation	0.04	0.07
Variance	0.0016	0.0049
95% Confidence Interval	[0.58, 0.64]	[0.78, 0.86]

**Table 6:** Statistical Significance Test (*t*-test Results).

Metric	t-Value	*p*-Value	Significance
Energy Consumption (*J*)	4.12	0.002	Significant
Latency	3.85	0.003	Significant
Network Lifetime	4.45	0.001	Significant
Remaining Energy	4.08	0.002	Significant

## Data Availability

Data supporting the reported results are available from the corresponding author upon reasonable request.
